# Autocrine/Paracrine Slit–Robo Signaling Controls Optic Lobe Development in *Drosophila melanogaster*

**DOI:** 10.3389/fcell.2022.874362

**Published:** 2022-07-25

**Authors:** M. Constanza González-Ramírez, Francisca Rojo-Cortés, Noemí Candia, Jorge Garay-Montecinos, Pablo Guzmán-Palma, Jorge M. Campusano, Carlos Oliva

**Affiliations:** Department of Cellular and Molecular Biology, Faculty of Biological Sciences, Pontificia Universidad Católica de Chile, Santiago, Chile

**Keywords:** nervous system development, cell segregation, axon guidance, slit–robo pathway, *Drosophila melanogaster*

## Abstract

Cell segregation mechanisms play essential roles during the development of the central nervous system (CNS) to support its organization into distinct compartments. The Slit protein is a secreted signal, classically considered a paracrine repellent for axonal growth through Robo receptors. However, its function in the compartmentalization of CNS is less explored. In this work, we show that Slit and Robo3 are expressed in the same neuronal population of the *Drosophila* optic lobe, where they are required for the correct compartmentalization of optic lobe neuropils by the action of an autocrine/paracrine mechanism. We characterize the endocytic route followed by the Slit/Robo3 complex and detected genetic interactions with genes involved in endocytosis and actin dynamics. Thus, we report that the Slit-Robo3 pathway regulates the morphogenesis of the optic lobe through an atypical autocrine/paracrine mechanism in addition to its role in axon guidance, and in association with proteins of the endocytic pathway and small GTPases.

## Introduction

The development of the nervous system requires a combination of specific cellular processes that occur in sequential but also overlapping manners, such as neurogenesis and axon guidance. While these processes are taking place, additional mechanisms prevent the intermingling of cells belonging to distinct compartments of the nervous system. These mechanisms of cell segregation might include the formation of specialized boundary cells or the interaction between cells at the interface between two regions (Kiecker and Lumsden, 2005; Dahmann et al., 2011; Batlle and Wilkinson, 2012; Fagotto, 2014; Addison and Wilkinson, 2016; Gonzalez-Ramirez et al., 2021). At the cellular level, the contribution of differential cell adhesion, cell repulsion, and differential interfacial tension have been well described (Batlle and Wilkinson, 2012; Fagotto, 2014). Notably, the signaling pathways upstream of these mechanisms are poorly characterized, and most research in this direction has focused on the Ephrin-Eph pathway (Cayuso et al., 2015; O'Neill et al., 2016; Wilkinson, 2021).

Cellular communication can occur through different mechanisms, depending on which cells secrete and/or receive the signals. When the ligand is secreted to the extracellular milieu and activates receptors in other cells, it is called paracrine signaling. On the other hand, when a cell secretes the ligand and also expresses the receptors, it is called autocrine signaling (Singh and Harris, 2005; Li et al., 2009). While paracrine signaling commonly regulates cell migration and axonal growth (Bashaw and Klein, 2010; Rorth, 2011; Yam and Charron, 2013), autocrine signaling plays important roles in stem cell biology and cancer (Sun et al., 2015; Zhou et al., 2018). Importantly, autocrine and paracrine signaling can occur simultaneously (Corriden and Insel, 2010).

Slit is a secreted protein, originally characterized by its participation in axon guidance in the ventral nerve cord (VNC) of the *Drosophila* embryo (Kidd et al., 1999). In this system, Slit, secreted by the midline glia, generates a concentration gradient that defines which neurons project their axons only on one side of the nervous system or will cross the midline forming commissures (Dickson and Gilestro, 2006). Slit also prevents the re-crossing of commissural axons and the positioning of the axon tracks parallel to the midline (Dickson and Gilestro, 2006). This mechanism is conserved in the spinal cord of vertebrates, where Slit is expressed in the floor plate (Brose et al., 1999). Slit elicits all these actions through Robo receptors, single-pass transmembrane proteins that modulate the organization of the growth cone, a structure that senses cues from the environment and is located at the tip of growing axons and dendrites (Dent et al., 2011; Franze, 2020; Lowery and Van Vactor, 2009; O'Donnell et al., 2009). In vertebrates, there are three Slit paralogues and four Robo receptors, whereas in *Drosophila* there is only one Slit and three Robo receptors, making it a simplified model for the study of this pathway (Dickson and Gilestro, 2006). Recent studies have shown that upon Slit binding to Robo, the complex is endocytosed by a clathrin-mediated mechanism and that Rab GTPases modulate the subsequent signaling (Chance and Bashaw, 2015). Downstream of Robo, several cytosolic signaling proteins can be activated leading to changes in the behavior of the growth cone, mainly through modifications of the cytoskeleton. Some of these mediators are Dock, Pak, Son of Sevenless (SOS), Vilse, and the vertebrate sr-GAP (Wong et al., 2001; Fan et al., 2003; Lundstrom et al., 2004; Yang and Bashaw, 2006; Lucas and Hardin, 2018).

Even in the nervous system, Slit and Robo receptors play roles beyond axon guidance. Recently, it was demonstrated that Slit–Robo is involved in cell segregation during fly optic lobe development (Tayler et al., 2004; Suzuki et al., 2016; Caipo et al., 2020); however, the downstream mechanisms remain elusive.

The optic lobe of *Drosophila* is formed by neuropils, in which visual information coming from the retina is processed. These neuropils include the lamina, which gathers information from the retina, and the medulla, which receives information from the retina and lamina and then sends it to the lobula complex formed by the lobula and the lobula plate. Information can be further integrated into the central brain, where it is processed by higher centers such as the central complex (Perry et al., 2017; Contreras et al., 2019; Courgeon and Desplan, 2019). The precursors of these four neuropils are already separated in the larval stage. In contrast to the VNC, in the fly optic lobe, Slit is expressed in rather diffuse patterns in several cellular populations, including glial and neuronal cells. Thus, although Slit also regulates the navigation of axons in the optic lobe (Pappu et al., 2011), it is unclear whether it does it through a graded signal, at least during larval stages (Tayler et al., 2004). Furthermore, it has also been recognized that Slit plays a role in optic lobe compartmentalization, since *slit* mutants exhibit ectopic cells between neuropils. Overall, the evidence suggests that Slit–Robo and also Netrin–Frazzled, another well-known axon guidance system, work together by counteracting mechanisms of attraction and repulsion to drive cell segregation (Suzuki et al., 2018).

Although the functions of Robo receptors have been addressed in the developing optic lobe, it is not clear whether different Robo paralogues mediate specific functions in *Drosophila*. In addition, the molecular mechanisms necessary to modulate cell behavior in this context have not been addressed.

In this work, we have shown that Slit and Robo3 constitute an autocrine/paracrine signaling pathway acting in medulla neurons of the optic lobe, and necessary for optic lobe neuropil segregation. *slit* and *robo3* mutants show strong defects in optic lobe morphogenesis, which are recapitulated by specific knockdowns in a subpopulation of medulla neurons. We also observe non-autonomous defects in photoreceptor axons that normally receive Slit from medulla neurons. Finally, we have demonstrated that this pathway is regulated by endocytosis and acts upstream of the cytoskeletal regulators Rac1 and Cdc42.

## Results

### Slit and Robo3 are Co-expressed in Medulla Neurons and are Required for Optic Lobe Development

**FIGURE 1:**
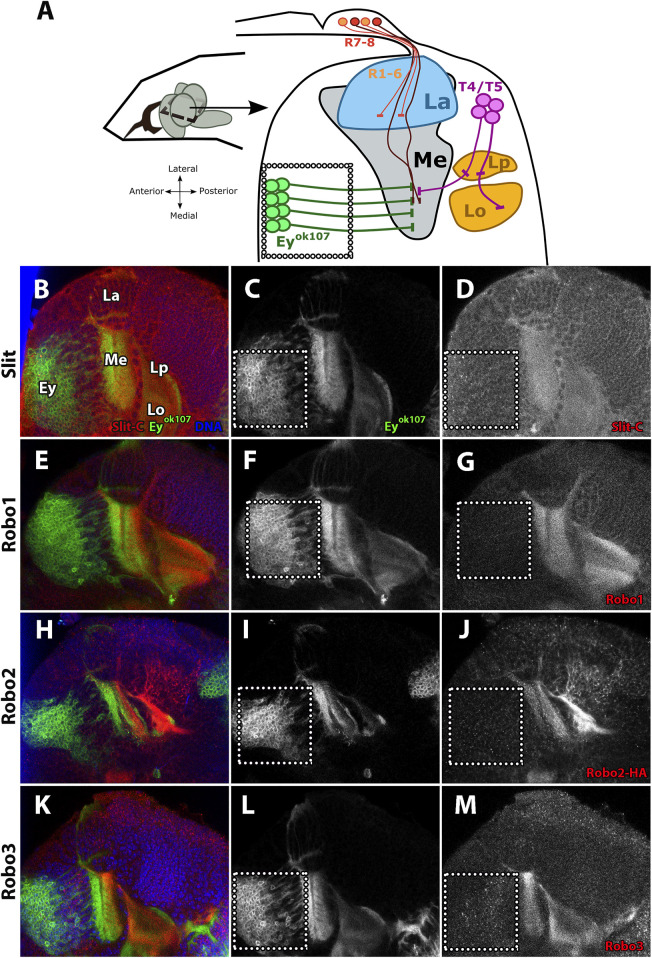
Slit and Robo expression patterns during the development of the visual system. **(A)** Diagram of the distribution of neurons in the L3 larval stage, horizontal view. R1–6 photoreceptors project their axon from the eye imaginal disc to the lamina (La), while R7–8 photoreceptors project them toward the medulla (Me). Perpendicular to the photoreceptor axons, the Eyeless (Ey+) neurons project their axons through the medulla. **(B–M)** Immunofluorescence of Slit and Robo (red) in an *ey* ^*OK107*^ *-GAL4* driving CD8-GFP (green) larva (L3 stage) showing the expression patterns in different developing neuropils. Ey + medulla neurons are delimited by the dotted line. **(B–D)** Expression pattern of Slit shows a homogeneous distribution and similar intensity in the medulla and lobula complex. **(E–G)** Robo1 expression pattern is similar to Slit expression. **(H–J)** With Robo2, we used Robo2-HA endogenously tagged (Spitzweck et al., 2010) for visualizing this receptor. Its expression pattern shows distribution in somas of T4/T5 neurons and high expression in the lobule complex, while weaker staining is observed in the medulla neuropil. **(K–M)** Robo3 is expressed in all optic lobe neuropils and shows a punctate distribution in somas of Ey + medulla neurons similar to Slit. La, lamina; Me, medulla; Lp, lobula plate; Lo, lobula. Schematic representation inspired by Caipo et al., 2020. *N* = 3 for all experiments. Single slice is presented. Scale bar: 30 μm.

It has been previously shown that Slit and the three *Drosophila* Robo receptors are expressed in the medulla neuropil in addition to other regions of the visual system (Tayler et al., 2004; Suzuki et al., 2016; Caipo et al., 2020; Guzman-Palma et al., 2021). Furthermore, in our previous work, we demonstrated that Ey + medulla neurons in the optic lobe are an important source of Slit (Caipo et al., 2020). To further characterize the expression of Slit and Robo receptors in the optic lobe, we performed immunostaining of the four proteins in the L3 stage and examined horizontal sections of the optic lobe, which allowed us to observe all neuropils in the same plane (Figures 1A-M). Interestingly, we noted that the Slit signal is detected in the same regions in which Robo receptors are expressed, suggesting that Slit and Robo receptors are co-expressed in the optic lobe (Figures 1D,G,J,M).

**FIGURE 2:**
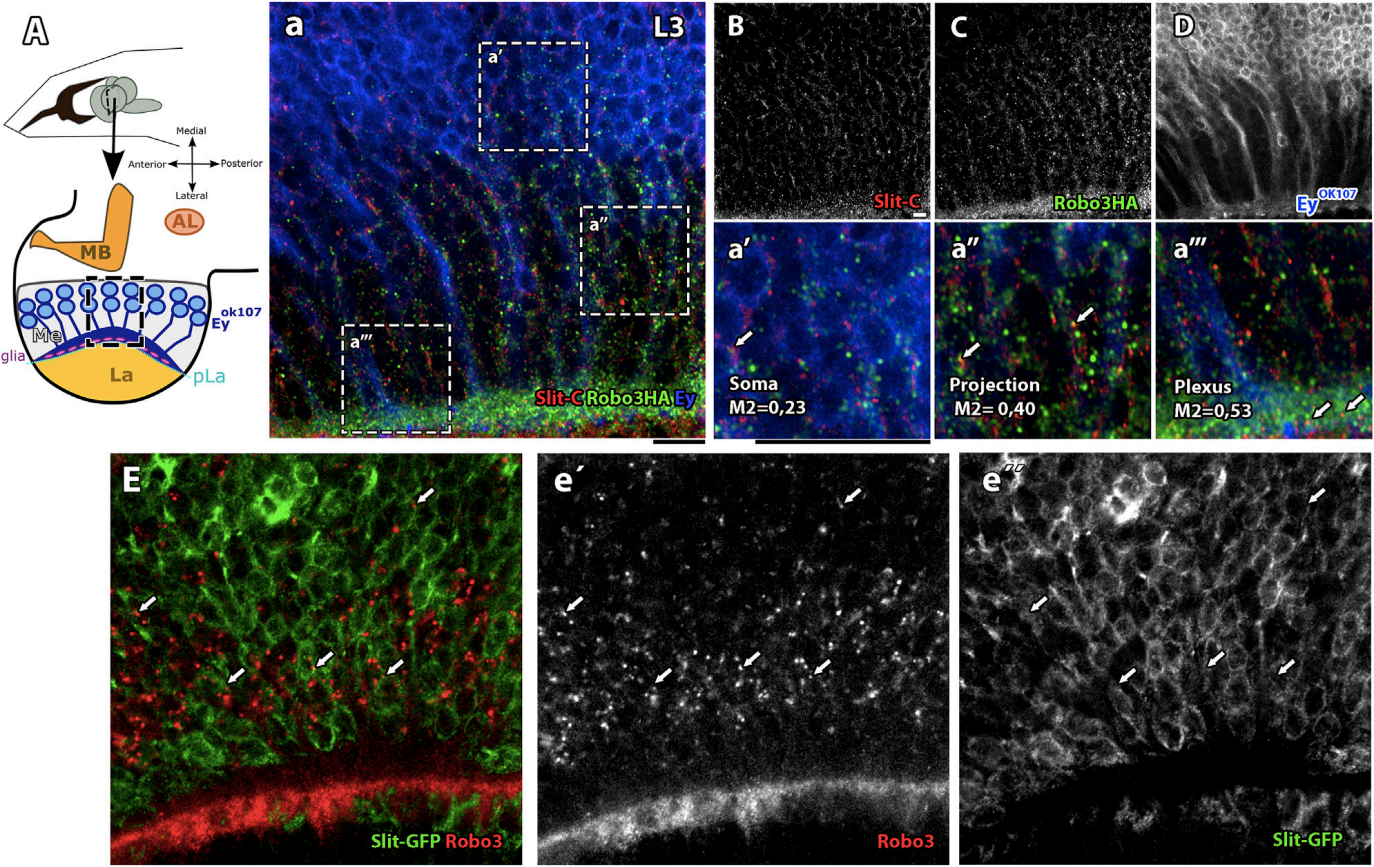
Slit/Robo3 co-localization in Ey + neurons in the developing visual system. **(A)** Diagram of Ey + neurons in the L3 larval stage, frontal view. The growth cones of Ey + medulla neurons are located in the medulla (Me) next to the Lamina plexus (pLa), which delimits the medulla and lamina. We labeled with anti-Slit and anti-HA (endogenously tagged, Robo3-HA (Spitzweck et al., 2010)). **(a–D)** Visualization of an area of the medulla shows that Slit and Robo3 have similar localization patterns and some punctate structures co-localize. The most enriched area for both proteins is next to the plexus region **(a```)** with Manders coefficient M1 = 0.53. There is also important co-localization in neuronal projections with M1 = 0.4 **(a``)** and in the Soma with M1 = 0.23 **(a`)**. N = 5. Single slice. **(E)** Slit-GFP reporter line labeled with anti-Robo3 (red, **e`**) and GFP (green, **e``**). Visualization of medulla area development shows Robo3 presence in Slit + cells (arrow). *N* = 3. Single slice. AL: antennal lobe and MB: mushroom bodies. All images have a scale bar = 15 μm.

The null mutant allele of the *robo3* gene, *robo3^3^* (Supplementary Figures S1A–D), has been previously characterized; it showed similar phenotypes to *slit* mutants in photoreceptor axons (Pappu et al., 2011). Due to the resemblance in the mutant phenotypes, we decided to study the relationship between Slit and Robo3 in medulla neurons. Examination of Slit and Robo3 expression in Ey + medulla neurons indicate that these two proteins co-localize in Ey + medulla neurons in a punctate pattern, especially in projections and the plexus region where growth cones are located (Figure 2 A–D, a`–a` ` `). In addition, using a Slit-GFP reporter (Figures 2E–e` `) (Plazaola-Sasieta et al., 2019; Caipo et al., 2020) to identify Slit-expressing cells, we confirmed that cells expressing Slit in the medulla neurons are also positive for Robo3 expression. These results support that Slit and Robo3 are co-expressed in medulla neurons, suggesting the possibility of an autocrine/paracrine pathway.

**FIGURE 3:**
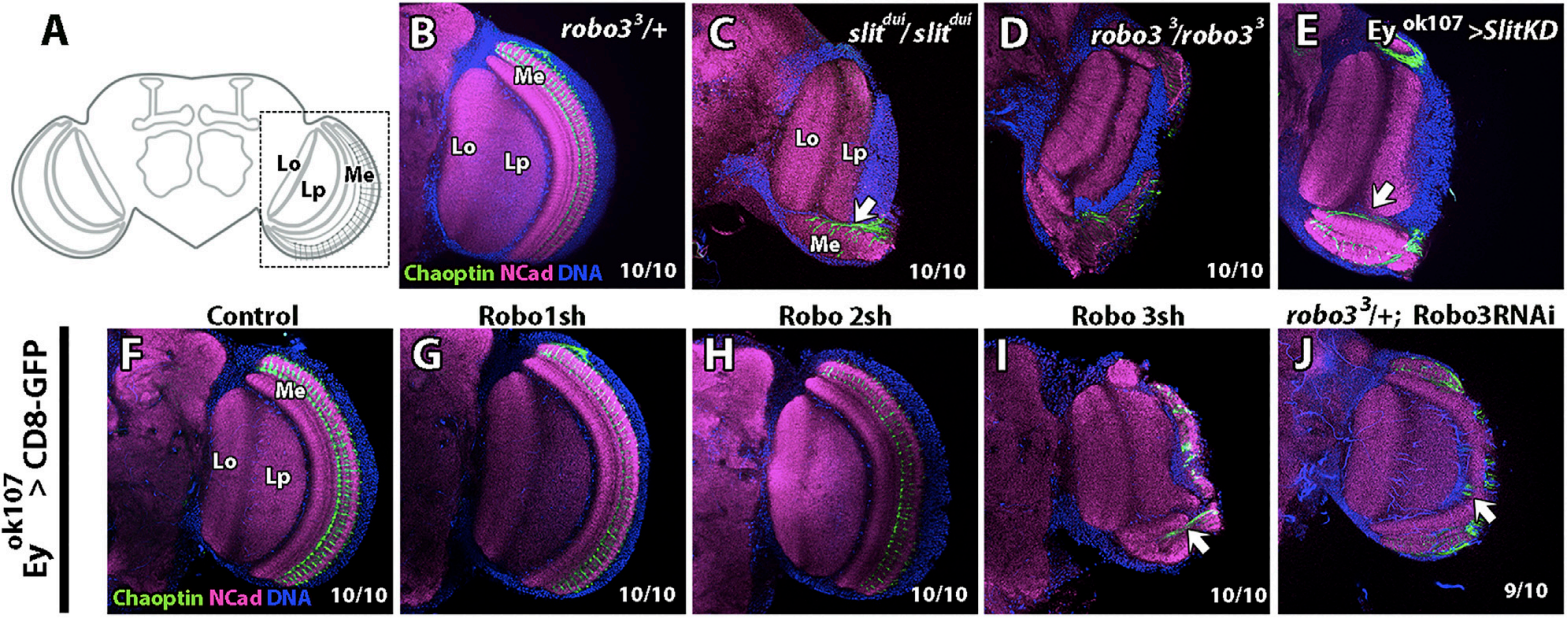
*slit* ^*dui*^, *robo3^3^*, and Robo knockdown phenotypes in the optic lobe. **(A)** Diagram of the fly adult brain focused on the optic lobe, frontal view. Medulla (Me), R7–8 photoreceptor axons, and lobula complex (Lo and Lp) is indicated. **(B)** Immunofluorescence of the adult stage using anti-Chaoptin (photoreceptors) and N-Cadherin (neuropil) shows that *robo3^3^* null mutation in heterozygosity displays a control phenotype. **(C)** *slit* ^*dui*^ hypomorphic mutant has a disrupted medulla and ectopic photoreceptor fascicules (arrow). **(D)** *robo3* ^3^ null mutation in homozygosity displays a similar phenotype. **(E)** Slit knockdown (KD) in Ey + cells show similar phenotypes as the *slit* or *robo* mutations previously described. **(F–J)** Phenotypes of Robos KD in Ey + neurons. Experimental control **(F)** as well as Robo1 KD **(G)** and Robo2 KD **(H)** show a wild-type phenotype. **(I)** Robo3 KD phenotype is similar to *robo3* and *slit* mutant animals. **(J)** Using another Robo3-RNAi line in a *robo3* mutant background heterozygote also show a disrupted medulla phenotype. *N* = 10 for all experimental conditions. All images are from single slices. Scale bar: 30 μm.

One of the most prominent phenotypes of *slit* mutants in the optic lobe is the intermingling of lobula complex cells with lamina and medulla neuropils during development (Tayler et al., 2004; Suzuki et al., 2016; Plazaola-Sasieta et al., 2019; Caipo et al., 2020), which leads to strong morphological defects in the adult optic lobe. A similar phenotype is observed when Slit is knocked down, specifically in Ey + medulla neurons (Caipo et al., 2020). In contrast to Slit, the cell-specific requirements of Robo receptors in the optic lobe are less characterized, although it was previously shown that knocking down the three Robo receptors simultaneously in all neurons produced a phenocopy of the *slit* mutant (Tayler et al., 2004). Studies in whole mutant animals showed that *robo2* and *robo3* mutants at the larval stage displayed similar boundary defects to those observed in *slit* mutants (Suzuki et al., 2016). In addition, we previously assessed the phenotypes of *robo2* mutants in the adult optic lobe, finding that they are subtler than the phenotypes of *slit* mutants in adult animals, and connected to their function in the lobula plate (Guzman-Palma et al., 2021). In the case of Robo3, we examined the adult optic lobe in *robo3^3^* mutants (Figures 3A–D) and noticed defects in neuropil organization that are similar to those observed in the *sli* ^*dui*^ mutant, which is a hypomorphic allele with decreased Slit expression, especially in the optic lobe (Figure 3E and Supplementary Figures S1E–H). In *robo3^3^* mutants, we observed strong medulla defects, in addition to the R-cell defects already reported. These results suggest that Robo3 plays a critical role in the development of the optic lobe particularly in the medulla. Next, we decided to test whether Robo3 or the other Robo receptors have autonomous roles in medulla neurons. We performed knockdowns using shRNAi with the GAL4/UAS system (Brand and Perrimon, 1993) in Ey + medulla neurons using the *ey* ^*OK107*^ *-GAL4* driver (Figures 3F–J) expressing previously tested RNAi lines in the optic lobe ((Guzman-Palma et al., 2021) Supplementary Figures S1I–Y) and analyzed the morphology of the adult optic lobe. We found that only the Robo3-RNAi knockdown (KD) produced strong alterations in optic lobe morphology, using two independent lines (Figures 3I,J), which is a consequence of the compartmentalization defect produced during development. These alterations are similar to those of *slit* and *robo3* mutants and to the Slit-KD in medulla neurons reported previously (Figures 3C-E) (Caipo et al., 2020). To better characterize the role of Robo3 in optic lobe development, we performed Robo3 KD experiments in photoreceptor and glial cells. We found that Robo3 is also important in photoreceptor cells since Robo3 KD led to strong medulla defects (Supplementary Figure S2A–D) and in glial cells where Robo3 KD led to subtler defects in photoreceptor axons (Supplementary Figure S2E–H). Notably, we also detected Robo3 expression in glial cells sitting on the lamina plexus (Supplementary Figure S3A–F). These results suggest that Robo3 is required in several cell populations for correct optic lobe compartmentalization.

In summary, our data show that Slit and Robo3 are co-expressed and required for the development of medulla neuron, and suggest that the mechanism involves autocrine/paracrine signaling at least in Ey + medulla neurons.

### Slit–Robo3 Signaling in Medulla Neurons is Regulated by Endocytosis

**FIGURE 4:**
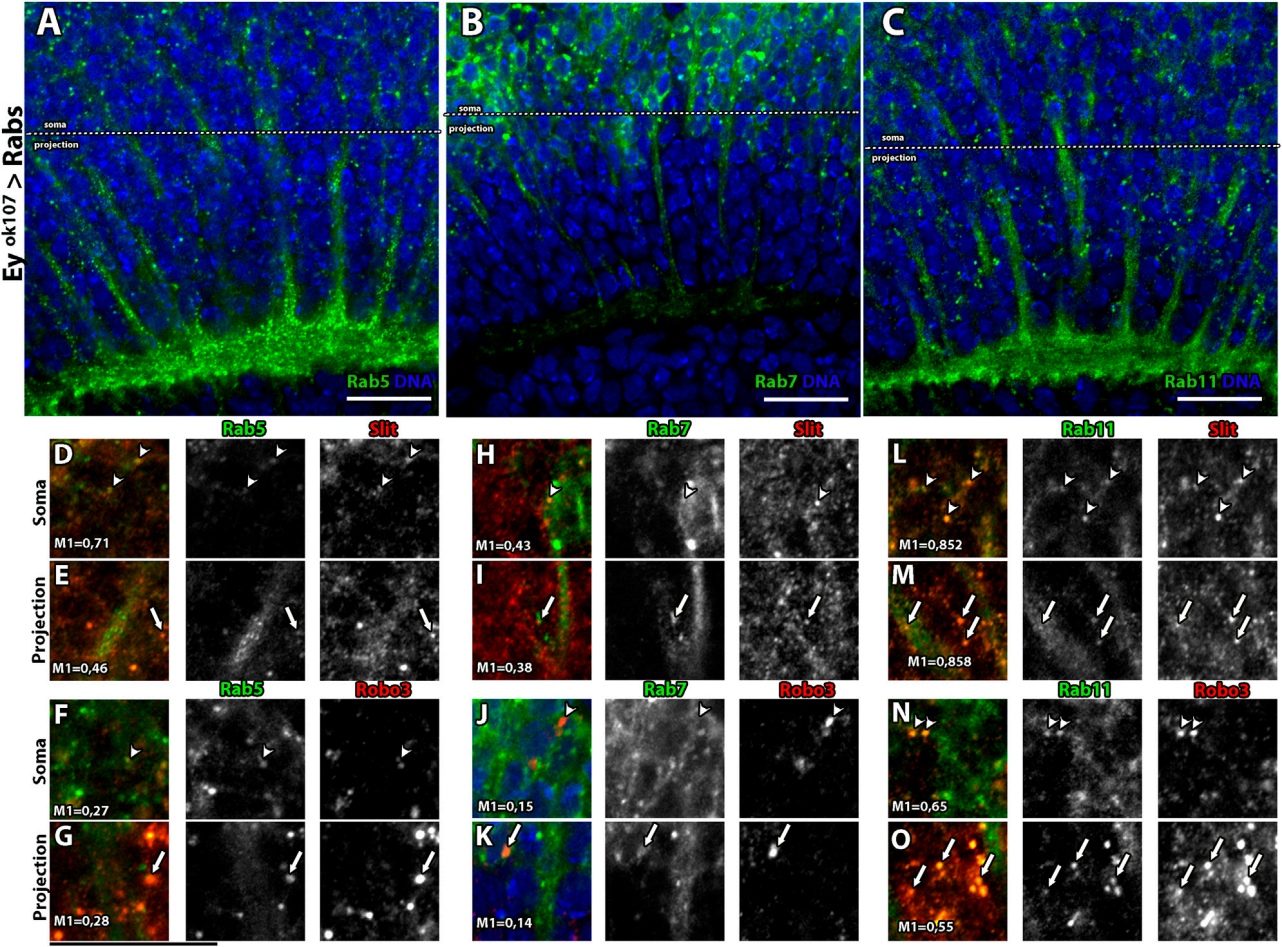
Rab GTPases co-localize with Slit and Robo3 in Ey + neurons *in vivo*. **(A–C)** Expression pattern of Rab5, Rab7, and Rab11 reporters tagged with GFP in Ey + neurons (larval stage) frontal view. For better visualization, Rab11-GFP and Rab7-GFP were labeled using anti-GFP. Rab5 and Rab11 are enriched in the axon growth cone (plexus region), while Rab7 is enriched in the Soma of Ey + neurons. **(D–O)** Slit and Robo3 immunofluorescence show different levels of co-localization with Rab proteins, indicated with arrow heads for somas and arrows for projections. Co-localization is presented using Manders coefficients. Rab5 has a higher level of co-localization with Slit in Soma (M = 0.71) **(D)** vs. projection (M1 = 0.46) **(E)**. Rab7 has similar levels of co-localization with Slit in Soma (M1 = 0.43) **(H)** and projection (M1 = 0.38) **(I)**. In the case of Rab11, high levels of co-localization with Slit are observed in both compartments (M1 = 0.85) **(L–M)**. On the other hand, Rab5 and Robo3 show similar levels of co-localization in Soma (M1 = 0.27) **(F)** and axon (M1 = 0.28) **(G)**. Rab7 shows low levels of co-localization with Robo3 in both compartments, soma (M1 = 0.15) **(J),** and projection (M1 = 0.14) **(K)**. Rab11 has the highest levels of co-localization with Robo3, M1 = 0.65 in the Soma **(N)** and M1 = 0.55 in the projection **(O)**. *N* = 5. All images are from single slices. Scale bar: 15 μm.

**FIGURE 5:**
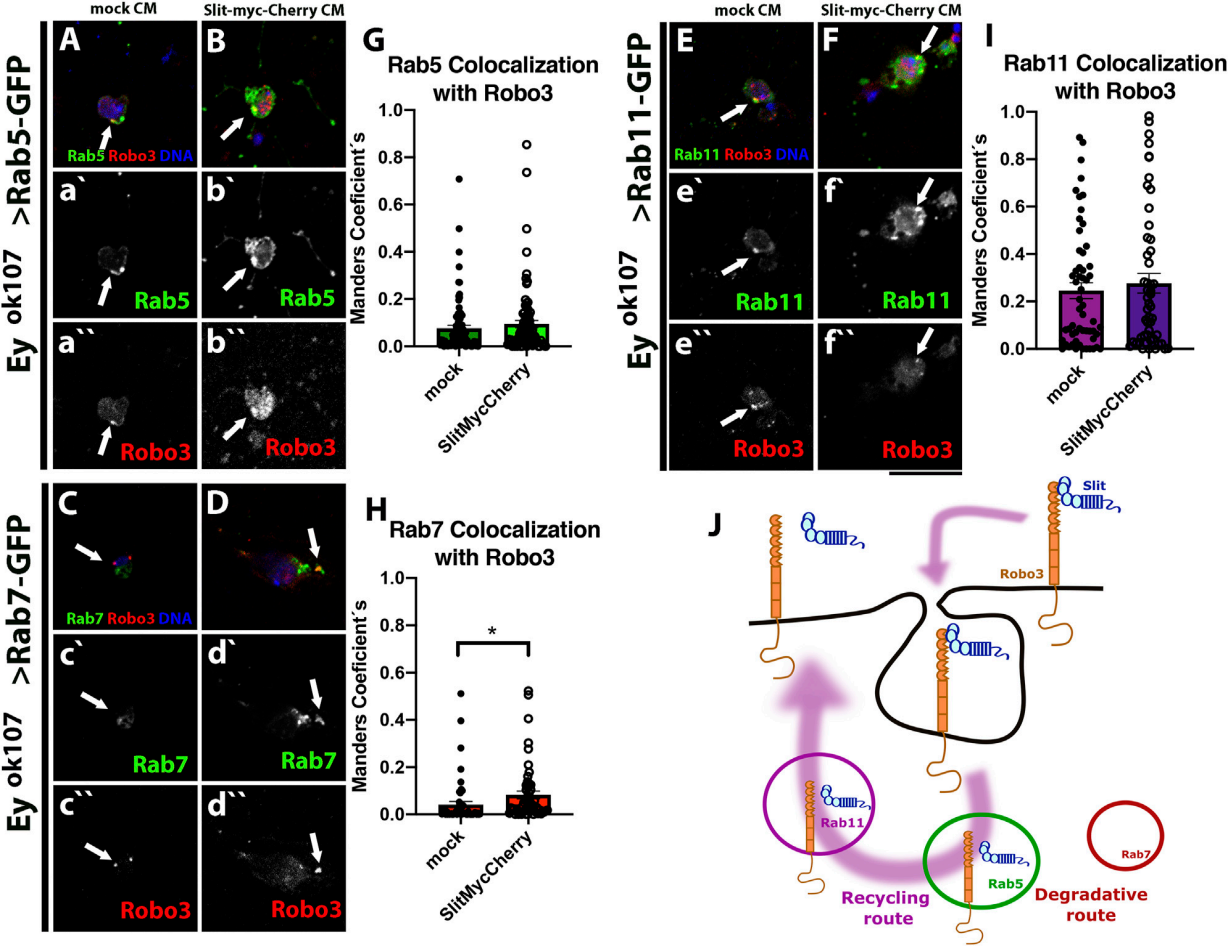
Robo3 co-localized with Rabs in Ey + neurons *in vitro.* Primary cell culture of Ey + neurons expressing Rab5-GFP (*N* = 5, **(A–B)**), Rab7-GFP (*N* = 4, **(C–D)**), and Rab11-GFP (*N* = 4, **(E–F)**) treated with mock or Slit-myc-Cherry conditioned medium (CM), respectively. Closed-up views: Robo3 (red), Rabs-GFP (green), and DNA (blue) are present in a`-f```. For staining the internalized protein, we performed an acidic wash and immunofluorescence against Robo3 (red). Arrows shows co-localization puncta. After 15 min treatment, Rab7-GFP shows higher levels of co-localization with Robo3 in the presence of Slit compared with the mock treatment **(H)**. Rab5 **(G)** and Rab11 **(I)** show no significant differences but there is a tendency toward increase co-localization with Slit-myc-Cherry treatment. **(J)** Schematic representation of Robo3 and Slit (information on Supplementary Figure S7) shows an endocytic recycling route. Error bar: Mean ± SEM. The Mann–Whitney test, *p < 0.05. Images are Z projections from two slices, Scale bar: 15 μm.

In recent years, several cellular mechanisms have been shown to collaborate with Slit–Robo for signal transduction. It has been reported that Slit-Robo1 signaling in the VNC of the *Drosophila* embryo is regulated by endocytosis (Chance and Bashaw, 2015). Interestingly, Robo3 also bears a predicted putative motif for clathrin-dependent endocytosis (not shown). Therefore, we assessed whether endocytosis and the post-endocytic trafficking play a role in the Slit/Robo3 autocrine/paracrine pathway using Airyscan confocal microscopy. First, we expressed GFP reporters for main Rab GTPases located in different types of endosomes (Zerial and McBride, 2001) using the *ey* ^*OK107*^ *-GAL4* driver (Figures 4A–C). We used Rab5 (early endosomes), Rab7 (late endosomes), and Rab11 (recycling endosomes). Importantly, the expression of these fusion proteins did not alter the morphology of the optic lobe (Supplementary Figure S4). We found that Slit and Robo3 are localized in all types of endosomes, especially in Rab5 and Rab11 positive endosomes and to a lesser degree in Rab7 positive endosomes (Figures 4D-O). However, the presence of Slit and Robo3 in endosomes could originate from anterograde trafficking after the process of protein synthesis. To confirm that sorting to endosomes can occur from the cell membrane upon endocytosis, we performed internalization assays using primary cell cultures of larval brains (Figures 5A–J) expressing the different Rab reporters under the control of *ey* ^*OK107*^ *-GAL4*. Cells were incubated with Slit-myc-Cherry obtained from a stably transfected *Drosophila* S2 cell line (Supplementary Figure S5). The Slit-myc-Cherry protein can be detected in S2 cell media (Supplementary Figure S5A–C), and its overexpression can disturb the development of photoreceptor axons (Supplementary Figure S6A). However, Slit-myc-Cherry expression in Ey + neurons could not rescue the *slit* mutant phenotype in the optic lobe (Supplementary Figure S6B), as previously observed for an untagged Slit protein (Caipo et al., 2020). These results indicate that this tagged version is less active or may have a lower rate of synthesis/secretion. We observed Robo3 in early (Rab5+), late (Rab7+), and recycling (Rab11+) endosomes 15 min after treatment with Slit (Figures 5G–I). Furthermore, incubation with Slit increased the co-localization of Robo3 receptors to late endosomes compared to the mock treatment (Figures 5A–D,G–H). Finally, we tested whether N-Slit (the Slit fragment that contains the Robo binding site) moves to endosomes from the cell membrane upon incubation with neurons. We observed that Slit is present in the three types of endosomes 15 min after incubation (Supplementary Figure S7 and Supplementary Figure S8). These results indicate that Slit and Robo3 can be endocytosed to enter the recycling route (Figure 5J).

### Sorting of Slit and Robo3 for Recycling Endosomes in Medulla Neurons is Required for Optic Lobe Development

**FIGURE 6:**
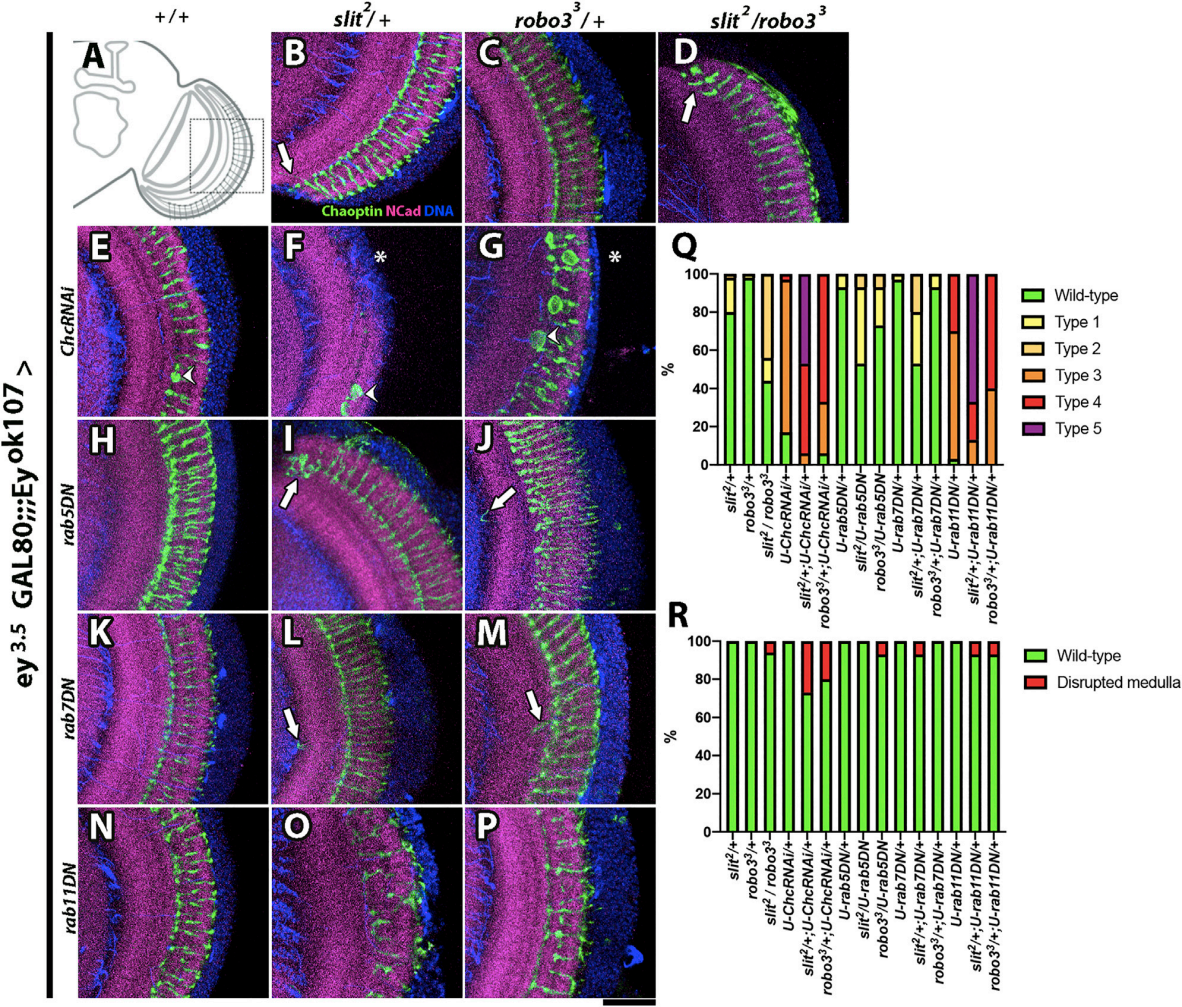
Genetic interaction between *slit*, *robo3*, *rab GTPases*, and *clathrin*. **(A)** Schematic representation of an optic lobe in the adult stage. Close-up frontal views of the medulla neuropil. Immunofluorescence against Chaoptin (photoreceptors, green), N-Cadherin (neuropils, magenta), and DNA (blue) of flies expressing *ey* ^*3.5*^ *-GAL80* and *ey* ^*OK107*^ *-GAL4*, which allows the expression of transgenes carrying the UAS promoter in Ey (+) medulla neurons, while repressing expression in the eye disc (Hoechst is included as counterstaining, blue). Dominant-negative (DN) forms of Rabs or RNAi for Clathrin were expressed. **(B)** *slit* ^*2*^ */+* experimental control shows some minor defects, consisting of occasional ectopic photoreceptor axons (arrow, *N* = 60). **(C)** *robo3* ^*3*^/+ has a wild-type phenotype (*N* = 60). **(D)** *slit* ^*2*^ */robo3* ^*3*^ shows ectopic photoreceptor axons (arrow, *N* = 16). **(E)** *ChcRNAi/+* flies display mild disorganization of photoreceptors, and it is the only GI that has a swelling axon (arrowhead, *N* = 30). **(F)** *slit* ^*2*^ */ChcRNAi* flies show a medulla disruption phenotype (*N* = 15), which is also seen in *robo3* ^*3*^ */ChcRNAi* **(G)**, asterisk, *N* = 15). **(H)** *rab5DN/+* (*N* = 30) and **(K)** *rab7DN/+* (*N* = 30) show wild-type phenotypes. **(I)** *slit* ^*2*^ */rab5DN* (*N* = 15). **(J)** *robo3* ^*3*^ */rab5DN* (*N* = 15). **(L)** *slit* ^*2*^ */rab7DN* (*N* = 15) and **(M)** *robo3* ^*3*^ */rab7DN* (*N* = 15) display ectopic photoreceptor axons (arrow). **(N)** *rab11DN/+* flies show mild photoreceptor disorganization (*N* = 30), while **(O)** *slit* ^*2*^ */rab11DN* (*N* = 15) and *robo3* ^*3*^ */rab11DN* **(P)**, N = 15) show strong photoreceptor disorganization and occasional disruption of the medulla. **(Q)** Graph shows the frequency of photoreceptor phenotypes evaluated: Wild-type, Type 1 (one ectopic photoreceptor axon), Type 2 (≥2 ectopic photoreceptor axon), Type 3 (mild disorganization of photoreceptors), Type 4 (strong disorganization of photoreceptors), and Type 5 (photoreceptor disorganized + ectopic axons). **(R)** Graph displays the frequency of medulla disrupted phenotypes. *N* = 15 for every GI experiment. Images are Z projections from five slices. Scale bar: 30 μm.

We performed genetic interaction experiments, in which RNAi or dominant-negative proteins for distinct components of the endocytic machinery were expressed in Ey + medulla neurons combined with heterozygotes for *slit* or *robo3* mutants (Figures 6A-R, Supplementary Table S1). We included an *ey* ^*3.5*^ *-GAL80* transgene to avoid the expression of the driver in the eye-antennal imaginal disc (Supplementary Figure S9), which could affect optic lobe development (Huang and Kunes, 1996). For these experiments, we focused on two qualitatively distinct phenotypes: the appearance of strong defects in the medulla neuropil, in which the medulla is disrupted or disorganized, and the alterations in photoreceptor axons, such as ectopic photoreceptors (see Supplementary Figure S10 for a detailed description), which are likely to be indicators of more subtle defects in medulla organization and/or defects in Slit availability in the extracellular milieu. In addition, we observed axonal swelling defects in photoreceptor axons when Clathrin was disturbed, which may reflect its involvement in additional signaling pathways. Therefore, we did not consider this phenotype in our quantification analysis (Supplementary Figure S10 **N–O ` ` `)**). We found strong genetic interactions of *slit* and *robo3* mutants with *clathrin* and *rab11* and milder defects with *rab5* and *rab7* (Figures 6Q–R, Supplementary Table S1). Thus, our results suggest that the Slit-Robo3 signaling pathway involves the participation of the endocytic machinery, including Rab11 and recycling endosomes. These data contrast with a previous study reporting strong interactions of Slit-Robo1 with Rab7 suggesting the participation of late endosomes (Chance and Bashaw, 2015).

### Slit-Robo3 Regulates Rac1 and Cdc42

**FIGURE 7:**
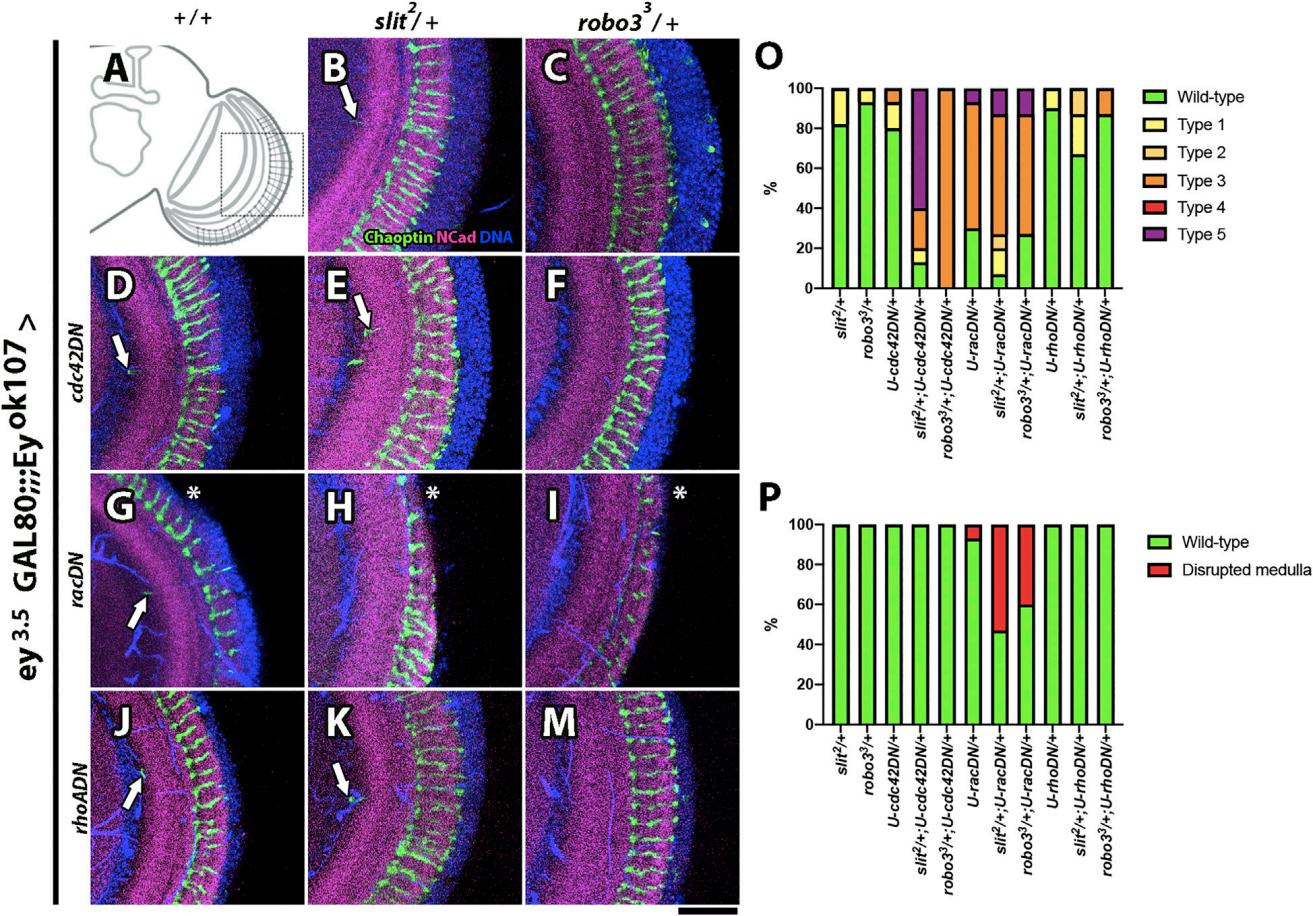
Genetic interactions between *slit*, *robo3*, and *Rho GTPases*. **(A)** Schematic representation of an optic lobe in the adult stage. Close-up frontal views of the medulla neuropil. Immunofluorescence against Chaoptin (photoreceptor, green), N-Cadherin (neuropils, magenta), and DNA (blue) in the *ey* ^*3.5*^ *-GAL80* and *ey* ^*OK107*^ *-GAL4* background. Dominant-negative constructs for small Rho GTPase proteins were expressed in Ey + medulla neurons as indicated. **(B)** *slit* ^*2*^ */+* shows few ectopic photoreceptor axons (arrow, *n* = 45). **(C)** *robo3* ^*3*^/+ flies show a wild-type phenotype (*N* = 45). **(D)** *cdc42DN/+* flies display ectopic photoreceptor axons (arrow, N = 30). **(E)** *slit* ^*2*^ */cdc42DN* flies display two or more ectopic photoreceptor axons (*N* = 15). **(F)** *robo3* ^*3*^ */cdc42DN* photoreceptor axons are disorganized (*N* = 15). **(G)** *racDN/+* presents an ectopic photoreceptor axon and disrupted medulla (asterisk, *N* = 30), but *slit2/racDN* (N = 15, **H**) and *robo3* ^*3*^ */racDN* (*N* = 15, **I**) flies show higher frequency of disrupted medulla phenotype. **(J)** *rhoADN/+* flies show ectopic photoreceptor axons (*N* = 30). **(K)** *slit* ^*2*^ */rhoADN* (*N* = 15) and **(M)** *robo3* ^*3*^ */rhoADN* (*N* = 15) display a similar phenotype to *rhoADN/+*. **(O)** Graph showing the frequency of photoreceptors phenotypes that were evaluated: Wild-type, Type 1 (one ectopic photoreceptor axon), Type 2 (≥2 ectopic photoreceptor axons), Type 3 (mild disorganization of photoreceptors), Type 4 (strong disorganization of photoreceptors), and Type 5 (disorganized photoreceptors + ectopic axons). **(P)** Graph shows the frequency of the disrupted medulla phenotype. Images are Z projections from five slices. Scale bar: 30 μm.

After establishing the participation of the endocytic pathway in Slit-Robo3 signaling, we assessed whether classical downstream targets of Robo1 in other contexts also take part in the process of medulla development (O'Donnell et al., 2009). We performed genetic interaction experiments with dominant-negative forms of the small GTPases Rac, RhoA, and Cdc42, and we scored the same phenotypes described before (see Supplementary Figure S10 for a detailed description). We found a strong genetic interaction of Slit-Robo3 with Rac1 and Cdc42 (Figures 7A-P, Supplementary Table S1), suggesting that these proteins are the main downstream effectors in this context.

## Discussion

In this work, we showed that a Slit-Robo3 autocrine/paracrine signaling pathway operates during the development of the medulla neuropil in the optic lobe of *Drosophila melanogaster*. As it has been shown in other contexts, this pathway may regulate the small GTPases Rac1 and Cdc42 (Chaudhari et al., 2021; Lundstrom et al., 2004; O'Donnell et al., 2009; Yang and Bashaw, 2006), which are likely to participate in the organization of the actin cytoskeleton to modulate cell segregation (Batlle and Wilkinson, 2012; Calzolari et al., 2014).

### Multiple Functions of Slit in Optic Lobe Development

The general concept of axon guidance cues in the nervous system is that a high concentration of the cue in one region will instruct the navigation of axons according to the repertoire of receptors in their membranes. However, we propose that the Slit gradient is not required for optic lobe morphogenesis. This work, as well as our previous work and reports from other labs, present several arguments in favor of this idea: 1) there is no gradient observed in the larval optic lobe; 2) the *slit* mutant phenotypes can be rescued by expressing Slit in various cell populations, such as medulla neurons and, partially, in glia and photoreceptor cells (Tayler et al., 2004; Caipo et al., 2020); and 3) large loss of function clones carrying the *slit* ^*2*^ allele in the visual system do not produce defects in optic lobe organization (Tayler et al., 2004). Indeed, Slit expression does not show a restricted source in the larval optic lobe, and previous data show that it is expressed in multiple cell types (Tayler et al., 2004; Caipo et al., 2020; Guzman-Palma et al., 2021), unlike in the central brain where it is enriched in the mushroom body (Oliva et al., 2016) and the VNC where it is expressed in the midline (Kidd et al., 1999; Dickson and Gilestro, 2006). Regarding the function of Robo receptors, the knockdown of all three Robo paralogues using a general driver resulted in defects in optic lobe development (Tayler et al., 2004). Here, we showed that the Robo3 function is required in medulla neurons since RNAi knockdown produces similar phenotypes as those observed in the *robo3* mutants. Robo3 is expressed in R8 photoreceptors where it regulates axon guidance, in response to Slit expressed in the optic lobe (Pappu et al., 2011). Thus, Slit performs two tasks during the development of the visual system. It serves as an axon guidance cue for photoreceptor axons (and perhaps other neurons) and it also prevents the intermingling of neighboring cell populations in the optic lobe.

### Slit–Robo Autocrine/Paracrine Signaling in Nervous System Development

In the nervous system, Slit and Robo receptors are largely expressed in complementary patterns (Kidd et al., 1998; Brose et al., 1999; Kidd et al., 1999; Dickson and Gilestro, 2006). Thus, in most contexts, Slit is secreted by a discrete group of cells, playing instructive roles for surrounding axons. Classic examples are the midline glia in insects and the floor plate in vertebrates, which play homologous functions in guiding commissural axons (Kidd et al., 1998; Brose et al., 1999; Kidd et al., 1999; Dickson and Gilestro, 2006). Few examples of Slit–Robo signaling playing an autocrine/juxtracrine role in the nervous system have been reported. In mouse, it can promote fasciculation of motor neurons (Jaworski and Tessier-Lavigne, 2012). In this case, Slit secreted by motor neurons binds to Robo1 and Robo2 in axons; it is necessary to avoid premature defasciculation at muscle targets. Another example is the co-expression of Slit2 and Robo2 during the development of Purkinje cells (Gibson et al., 2014). The deletion of either of these proteins leads to excessive dendrite self-crossing, demonstrating the role of this pathway in self-avoidance. One question is whether the downstream effectors are the same in these contexts. Regarding fasciculation, it is likely that the downstream effectors are adhesion molecules, such as cadherins, which are regulated by Robo in several different contexts (Rhee et al., 2002; Rhee et al., 2007; Guzman-Palma et al., 2021). Here, we find that in this context, regulators of the actin cytoskeleton are likely to be the downstream effectors of Robo3 in the optic lobe, although more work is required to unravel the complete mechanism.

### Boundary Formation in the Optic Lobe of *Drosophila*

The current model of neuropil compartmentalization in the optic lobe largely depends on the interplay between cell repulsion and attraction (Suzuki et al., 2018), in which complementary populations express ligands and receptors. Our data shows that this view has to be complemented based on the co-expression of Slit and Robo in at least some cellular populations. One interesting possibility is that autocrine/paracrine Slit-Robo3 signaling regulates the formation of actomyosin fibers that restrict cell movement from neighboring neuropils, leading to tissue separation. This mechanism has been extensively documented in vertebrates (Kiecker and Lumsden, 2005; Calzolari et al., 2014; Addison and Wilkinson, 2016) and also observed in other *Drosophila* tissues during development (Monier et al., 2010). RhoA is generally recognized as the GTPase that promotes the formation of actomyosin fibers. As shown here, recent literature also supports the participation of Rac and Cdc42 (which interact genetically with Slit in optic lobe development) in the initial steps of actomyosin fiber formation (Arnold et al., 2017). Another possibility is the regulation of cell repulsion, in which Slit binds Robo3 in the medulla and other Robo receptors (or a combination of them) in the lobula complex and lamina, which can promote disruption of cell–cell contacts at the interface between neuropils. This mechanism could be similar to the one involved in the separation of ectoderm and mesoderm in the early frog embryo, which depends on two antiparallel Eph-ephrin signaling processes (Wilkinson, 2021) triggered by both tissues (Rohani et al., 2011). Interestingly, one of the downstream effectors found in this study is Rac1, which can rescue the absence of Eph-Ephrin signaling in these tissues. One point in favor of this alternative is its compatibility with developing axons and dendrites undergoing migration in both neuropils using one another as substrate, which also happens in the interface between ectoderm and the migrating cells from the mesoderm.

### Participation of Endocytosis in the Slit–Robo Pathway

The role of endocytosis is currently recognized as an important factor in regulating several signaling pathways (Bokel and Brand, 2014; Cosker and Segal, 2014). In the case of the Slit–Robo pathway, only a few publications have explored this aspect during the development of the fly nervous system. Chance and Bashaw (2015) found that endocytosis was important for the function of Slit-Robo1 in VNC axon guidance in the fly embryo. In contrast to our data on Robo3, the authors found strong genetic interactions with mutants of Rab7 indicating a major role of the late endosomes, while we found that Rab11 and presumably recycling endosomes, may have a main role in optic lobe development. Resensitization could explain the importance of recycling, in which receptors are trafficked back to the cell membrane (Roosterman et al., 2004; Hinkle et al., 2012; Kharitidi et al., 2015). This explanation is in line with results reported in mice in which the GTPase Arf6 regulates Robo1 membrane availability to increase the repulsion of post-crossing axons during spinal cord development (Kinoshita-Kawada et al., 2019). Arf6 promotes the sorting of Robo1 to recycling endosomes, and the authors also observed functional interactions with Rab11 proteins in their primary culture experiments. Why is recycling important in the case of the optic lobe but not as crucial in the midline as shown by Chance and Bashaw? A possible explanation is that the levels of Slit in the optic lobe are lower than those in the embryo VNC (see Tayler et al., 2004, Figure 4A and Oliva et al., 2016; Figures 1D,E). Therefore, a continuous supply of receptors to the membrane may be required for adequate levels of signaling. Interestingly, the transfer of receptors to recycling endosomes can be favored by a low concentration of the ligand in some systems (Roosterman et al., 2004).

### Downstream Effectors of Robo3 Receptor in the Optic Lobe

Since Robo receptors are interchangeable in the optic lobe (Pappu et al., 2011), the downstream target activated in this context must share signaling molecules. The actin cytoskeleton is a common target for Slit–Robo signaling, which is conserved across evolution. Regulators of actin polymerization such as small Rho GTPases are downstream of Robo receptors in *C. elegans*, flies, and vertebrates (O'Donnell et al., 2009).

The cytoplasmic tails of all Robos have some conserved regions known as the CC domains. *Drosophila* Robo1 has four of these domains (CC0-3) but Robo2 and Robo3 have only CC0-CC1(Dickson and Gilestro, 2006). Most of the identified downstream effectors of Robo1 have been described. However, little is known about Robo2 and Robo3 effectors. For instance, several Robo1 effectors are recruited using CC2 and CC3 domains, such as SOS and Pak, which can activate Rac1 and Cdc42 (Fan et al., 2003; Yang and Bashaw, 2006). Since Robo3 is lacking CC2-3, it is still unclear how it may activate Rac1 and Cdc42, and therefore more work is required in this direction.

## Methods

### Fly Husbandry

Flies were raised on standard fly food at 25°C for an expression pattern analysis, genetic interaction, Rabs-GFP phenotype, and co-localization experiment. Slit-myc-Cherry overexpression and knockdown experiments using RNAi were performed at 29°C. UAS-Slit-myc-Cherry expressing flies were generated by BestGene Inc., United States. All other lines were obtained or generated using fly strains from the Bloomington Drosophila Stock Center (Bloomington, Indiana). The details of genotypes used throughout this work are presented in Supplementary Table S2.

### Slit-Myc-Cherry Construct

The Slit isoform C was used (FlyBase) for the design of the Slit-myc-Cherry construct. A sequence coding for a myc epitope (EQKLISEEDL), flanked by two Ig2 linker sequences (IASKPKGASVRA), was inserted at the end of the fifth EGF-like repeat, before the cleavage site (PDDYTGKYCEGHNMISMMYPQTSP). The stop codon was removed and the sequence coding for mCherry was inserted after an Ig2 linker sequence. The complete sequence was codon-optimized and KpnI restriction sites were added flanking the sequence. The construct was synthesized and cloned into a pUAST-attb vector in the KpnI site by GENEWIZ Inc., United States.

### Immunofluorescence

Larval L3 and adult stage brains were dissected using standard procedures (Wu and Luo, 2006). In brief, brains were dissected and fixed in 4% formaldehyde in 1X PBS for 20 min at room temperature. Then, samples were washed six times in PBT (0.3% Triton X-100 in 1X PBS) and blocked in 1% NGS/PBT for 30 min at room temperature. Primary antibodies were diluted in PBT and samples were incubated overnight at 4C. The next day, samples were washed six times with PBT, incubated with fluorescent-dye conjugated secondary antibodies for 2 hrs, and washed six times in PBT. Then, samples were incubated with Hoechst (Thermo Fisher Scientific) in PBT for 10 min and washed three times with PBT. Finally, samples were incubated for 1 h in 50% glycerol/PBS at 4°C. Samples were mounted using Vectashield mounting medium (Vector). Primary monoclonal antibodies obtained from Developmental Studies Hybridoma Bank are mouse anti-Slit (C55.6D; 1:10), mouse anti-Robo1 (13c9; 1:50), mouse anti-Robo3 (14c9, 1:50), mouse anti-Chaoptin (24B10; 1:10), and rat anti N-Cadherin (DN-Ex #8; 1:10). Other antibodies used were mouse anti-myc (9e10; Santa Cruz 1:100), rabbit anti-HA (c29f4; Cell Signaling 1:100)), rabbit anti-GFP (A6455 Invitrogen, 1:100), and anti-Cherry (632,543, ClonTech Laboratories; 1:1,000). Fluorescent-dye conjugated secondary antibodies were obtained from Jackson Immunoresearch (Pennsylvania, United States) and Invitrogen (Massachusetts, United States) and used 1:200.

### Imaging and Co-localization Analysis

For expression pattern analyses, images of the larval brain were acquired using a Z-step size of 1.5 μm. Adult optic lobe stacks were acquired using a Z-step size of 0.8 μm. For adult optic lobes, the images shown are Z projections or a single slice (indicated in figure legends). Images of larval primary cell cultures were acquired using a Z-step size of 0.5 μm. All images were acquired using an Airyscan confocal microscope (Zeiss) at the UMA PUC facility.

Co-localization analyses were performed using the Jacob plugin of Fiji in which Manders M1 represents channel 1 (Rabs) co-localized with channel 2 (Slit or Robo3). For *in vivo* co-localization of Rab proteins with Robo3 and Slit (*n* = 5 optic lobes), stacks with a Z-step size of 0.5 μm were acquired. For *in vitro* experiments, co-localization of Rab5 with Robo3 (*n* = 5) and Rab7 and Rab11 (both *n* = 4), 12–21 cells were used per sample. For co-localization of Rab proteins with N-Slit (anti-myc) 7–10 cells were used per sample (*n* = 2 for Rab5; *n* = 3 for Rab7 and Rab11). For *in vitro* experiments using cell culture, stacks were acquired with a Z-step size of 0.5 μm, and the representative images were generated using a Z projection of two sections in the middle of the stack.

For co-localization analyses of Slit and Robo3-HA (endogenously tagged) in the larval stage *n* = 5, Slit was acquired in channel 1 and HA in channel 2, and for graphs Manders M2 value was used. For co-localization experiments, a statistical analysis was performed using the Mann–Whitney non-parametric test, using Prism 8 software.

### Phenotypic Analysis

For a phenotypic analysis of mutant and RNAi-expressing animals, the sample sizes were *robo3^3^*: *n* = 10 and Robo KD: *n* = 10. In the case of genetic interaction experiments, *n* = 15 in all experimental conditions (an independent set of control flies was used in each experiment). The evaluated phenotypes of genetic interaction experiments are schematized in Supplementary Figure S10. For Rab proteins, overexpression experiments, *n* = 7; overexpression of Slit-myc-Cherry in adult stage using *GMR-GAL4*, *n* = 10.

### S2 Stable Transfection and Mock/Slit-Myc-Cherry Conditioned Medium Production

Stable S2 cells transfection was performed following the instruction of Thermo Fisher Scientific Manual 0000656 rec B0 catR69007. Four days after transfection, culture medium was replaced by Schneider insect medium supplemented with 7% fetal bovine serum (7% FBS, Biological Industries) and 300 μg/ml hygromycin (1,068,701, Invitrogen) and plated in a 96 well plate. After 3 days, the best clone was selected using an epifluorescence microscope to start its expansion. S2-transfected cells with mock (including pUAST empty vector + actin-GAL4 +pCoHygro (resistance vector)) or Slit-myc-Cherry (including pUAST-Slit-myc-Cherry + actin-GAL4 +pCoHygro) were plated using the Schneider medium. The Slit-myc-Cherry or mock conditioned mediums was collected 48 h after plating the medium, and this step was repeated three times every 24 h. The medium was centrifugated to remove cells and concentrated using an Amicon ultra-15 of 100KDA filter (Millipore). Western blot assays were performed to confirm the presence of Slit-myc-Cherry in the S2 cells and the conditioned medium. S2 cells were lysed using 100 μl of lysis buffer (20 nM HEPES, pH7.5, 100mM KCl, 5% glycerol, 10 mM EDTA, 0.1% Triton X-100, 1 mM DTT, and protease inhibitors). Samples of S2 mock and Slit-myc-Cherry (50 µg of total protein) and 10 µl of the concentrate conditioned medium were heated at 95°C for 5–10°min and loaded in 7.5% SDS-PAGE gels. The membrane was incubated overnight at 4°C with mouse anti-myc antibody 1:500 (Santa Cruz) diluted in blocking solution (5% milk in 0.1% Tween 20 PBS 1X). The secondary antibody was incubated for 2 h at room temperature in the blocking solution. A chemiluminescence reaction was performed using a WESTAR Supernova reagent (XLS3,0100 Cyanagen) and acquired using the UVITEC imaging system.

### Primary Neuronal Cell Culture

A primary cell culture was performed according to (Sicaeros et al., 2007) but using the larval L3 developmental stage. Larvae were rinsed with 70% ethanol followed by two more rinses in distilled water. Larval brains were dissected in dissecting solution (DS: 6.85 mM NaCl Na_2_HPO_4_, 0.001 Mm KH_2_PO_4_, 0.2772 mM HEPES, pH 7.4), and briefly spun. Then, brains were treated with papain (LS 03126, Worthington) for 30 min at room temperature. Samples were washed three times with a DMEM/F12 culture medium (12,400–016, Gibco) supplemented with 100 μg/ml Apo-transferrin, 30 nM selenium, 50 μg/ml insulin, 2.1 mM 20-hydroecdysone, 20 ng/ml progesterone, 100 μM putrescine (all from Sigma Aldrich), and 1% antibiotic/antimycotic (15,240,062, Gibco). The tissues were then mechanically disaggregated and mounted on Laminin/Concavalin-coated coverslips in the presence of DMEM/F12-supplemented medium. Cells obtained from two larval brains were used for each coverslip. The following day, conditioned media obtained from astrocytes cultured in the neurobasal medium supplemented with B27 (CNBM/27) was added to the cells. On the fifth day *in vitro* of the experiment, samples were processed for internalization assays.

### Internalization Assay

Primary cells culture of eyeless expressing neurons, bearing Rabs-GFP transgenes were incubated in 50% of mock CM or Slit-myc-Cherry CM for 15 min at 25°C. Cells were washed with DMEM/F12-supplemented medium at 4°C and then with acidic pH 3.6. Then, cells were fixed with 2% formaldehyde in 4% sucrose–PBS 1X for 20°min, followed by incubation with 0.15 M glycine for 15 min. For immunofluorescence, the primary antibodies used were mouse anti-Robo3 or rabbit anti-myc. The secondary antibody was obtained from Invitrogen (used 1:200) and the mounting medium used was Fluoromount-G™ (17,984–25, electron microscopy sciences).

## Data Availability

The raw data supporting the conclusion of this article will be made available by the authors, upon reasonable request.

## Supplementary Material

Supplementary Figure S1 Expression of Slit and Robo in *slit* ^*dui*^, *robo3^3^* mutants, and Robos KD animals. Immunofluorescence of Slit or Robo proteins in different experimental conditions. **(A,C)** Control Robo3 staining. **(B,D)** *robo3* mutant shows the absence of Robo3 staining. **(E,G)** Control Slit staining. **(F,H)** *slit* mutant shows a decrease in Slit staining compared to the control. Image are Z projection from five slices *N*=3. **(I–Y)** Robo KD in Ey+ neurons. Robo1sh and Robo3sh decreased the Robo1 and Robo3 staining compared to control, respectively (Robo1 I-L and Robo3 Q-T); in the case of Robo2, there is no difference of staining between control and Robo2sh observed **(M–P)**. Robo3-RNAi (long dsRNA) in a *robo3* mutation background shows decreased Robo3 staining compared to the control **(U–Y)**. Images are Z projections from five slices. N=3 for Robo1sh and *N*=4 for the other Robo–RNAi conditions. Scale bar: 30 μm.

Supplementary Figure S2 Robo3 knockdown in different cell populations of the optic lobe. Immunofluorescence of photoreceptor axons (Chaoptin, green) and neuropil (N-Cadherin, magenta) for visualizing the adult optic lobe phenotypes with Robo3 knockdown, frontal view. **(A)** Robo3 KD in photoreceptor cells leads to axon guidance defects and disrupted medulla phenotypes *N*=10/10. **(B–D)** Close-up visualization. **(E–H)** Robo3 KD in glial cells leads to mild defects, showing ectopic photoreceptor axons (one or more) *N*=7/10 and subtle defects in the medulla (arrow heads). Images are Z projections from five slices. Scale bar: 30 μm.

Supplementary Figure S3 Robo3 is present in optic lobe glial cells. **(A–C)** Immunofluorescence of Robo3 (green) in flies which carry a membrane RFP reporter under the control of the glial driver Repo-GAL4 (red), DNA (blue), and frontal views. **(D–F)** Close-up visualization in the medulla shows that Robo3 co-localizes with the glial cells (arrows). *N*=3. Images are from single slices. Scale bar: 30 μm.

Supplementary Figure S4 Overexpression of Rab proteins in Ey(+) neurons does not affect optic lobe development. **(A–P)** Immunofluorescence of adult optic lobes (frontal views) stained against Chaoptin (photoreceptor axon, green), N-Cadherin (neuropil, magenta), and DNA (blue). **(A)** Wild-type optic lobe. A close-up is shown in **(B–D)**, and normal optic lobe architecture is observed. **(E–H)** Rab5-GFP, **(I–L)** Rab7-GFP, and **(M–P)** Rab11-GFP expression under the control of the *ey^OK107^-GAL4* driver show the same phenotype as control flies. *N*=7 for all experimental conditions. Images are Z projections from five slices. Scale bar: 30 μm.

Supplementary Figure S5 Characterization of the Slit-myc-Cherry construct and N-Slit co-localization with Rabs in Ey+ neurons in vitro. **(A)** Schematic representation of the Slit-myc-Cherry construct. The Slit structure is composed of four leucine-rich repeats (D1–4, gray), seven EGF repeats (green), one Agrin-Perlecan-slit (AG, orange), and a Cysteine-knot (star shape). This construct has two different tags, which allow us to monitor the two fragments of Slit after cleavage. N-Slit has a myc tag (between EGF repeats, dark green) and C-Slit has a Cherry tag (located after the Cysteine-knot, red). **(B)** Stable transfection of the empty vector (mock) or Slit-myc-Cherry vector constructs in S2 cells. Cherry is observed in Slit-myc-Cherry expressing cells. **(C)** Western blot of anti-myc for S2 mock cells, S2 Slit-myc-Cherry cells, mock CM, and Slit-myc-Cherry CM. The Slit-myc-Cherry lysate and CM present two bands, and no signal is observed in mock condition. **(D)** Primary cell culture of Ey+ neurons expressing GFP treated with Slit-myc-Cherry CM for different exposition times to determine the optimal slit internalization time. Scale bar: 15 μm. **(E)** Expression of Slit-myc-Cherry under the control of *ey^OK107^-GAL4* shows the localization of N-Slit and C-Slit fragments. Single slice. Scale bar: 30 μm.

Supplementary Figure S6 Evaluation of Slit-myc-Cherry construct function. **(A)** Expression of Slit-myc-Cherry in photoreceptors using *GMR-GAL4* in the adult stage leads to photoreceptor axon mistargeting defects in the medulla (arrows). **(B)** Slit-myc-Cherry rescue experiment in Slit mutant background shows that the construct does not rescue the phenotype. The optic lobe shows disruption of the medulla with different penetration levels and photoreceptor axon mistargeting. Images are from single slices. Scale bar: 30 μm.

Supplementary Figure S7 Co-localization of N-Slit with Rab-GFP reporters in cell culture. Primary cell culture of Ey+ neurons expressing Rab5-GFP (*N*=2, **(A)**), Rab7-GFP (*N*=3, **(B)**), and Rab11-GFP (*N*=3, **(C)**) incubated with Slit-myc-Cherry conditioned medium (CM). For staining of the internalized protein, we performed an acidic wash and immunofluorescence against the myc tag. After a 15 min treatment, Slit internalization can be observed in these cells. The Cherry tag could only be detected using immunofluorescence (anti-Cherry). The highest co-localization is between Rab11 and N-Slit with M1=0.24, followed by Rab7 with M1=0.12. Rab5 shows the lowest co-localization with M1=0.01 probably due to the timing of the experiment. **(E,F)** Graph shows the degree of co-localization against the background (mock medium). Error bar: Mean ± SEM. Mann–Whitney test, **p* < 0.05. Images are Z projections from two slices. Scale bar: 15 μm.

Supplementary Figure S8 Ey+ Rabs-GFP primary culture, whole field images. Whole image field of the immunofluorescence for Robo3 (red, **(A–F)**) and N-Slit (red, **(G-I)**) internalization experiment, co-localization after 15 min of mock CM or Slit-myc-Cherry CM. Rabs-GFP transgenes are expressed only in Ey+ neurons (green). DNA staining (blue) shows that there are other cells in the field that do not express the driver. The analyzed cells are delineated by dotted line squares. Images are Z projections from two slices. Scale bar: 15 μm.

Supplementary Figure S9
*ey^3.5^-GAL80* suppresses the expression of *ey^OK107^-GAL4* in the eye imaginal disc. **(A)** Optic lobe from larva L3 stage labeled with CD8-GFP under the control of *ey^OK107^-GAL4*. Ey+ neurons, eye imaginal disc, and mushroom bodies are observed. **(B)** Using *ey^3.5^-GAL80* in the same genetic background, it is possible to block the expression in the eye imaginal disc with little effect on other tissues. **(A,B)** Single slice image. Scale bar: 30 μm. **(C)** Eye of adult flies expressing the *Rab11DN* transgene shows a strong morphological defect. No eye defects are observed in the presence of *ey^3.5^-GAL80* **(D)**. **(E)** No defects of photoreceptor (Chaoptin, green) and neuropil (N-Cadherin, magenta) are observed in *ey^3.5^-GAL80* flies expressing the same transgene. Images are Z projections from five slices. Scale bar: 30 μm.

Supplementary Figure S10 Genetic interaction phenotype representation. **(A)** Schematic representations of photoreceptor phenotypes observed in genetic interaction phenotypes showed in Figure 6, Figure 7: Wild-type, Type 1 (one ectopic photoreceptor axon), Type 2 (≥2 ectopic photoreceptor axons), Type 3 (mild photoreceptor disorganization), Type 4 (strong photoreceptor disorganization), and Type 5 (photoreceptor disorganization + ectopic axons). **(B–M´)** Representative Z projections from five slices for each phenotype. A close-up of photoreceptor distribution and medulla disruption are included in each case. **(N–N´)** Example of axonal swelling phenotype observed in experiments using *Chc-RNAi*. **(O´–O´´´)** Close up visualization of different slices show a swelling photoreceptor axon. This phenotype was not evaluated in IG. Scale bar: 30 μm.
